# Infrared Macrothermoscopy Patterns—A New Category of Dermoscopy

**DOI:** 10.3390/jimaging9020036

**Published:** 2023-02-06

**Authors:** Flavio Leme Ferrari, Marcos Leal Brioschi, Carlos Dalmaso Neto, Carlos Roberto de Medeiros

**Affiliations:** Medical Thermology and Thermography Specialization, Sao Paulo University Medicine School Clinical Hospital, São Paulo 01246-903, SP, Brazil

**Keywords:** thermography, infrared spectrophotometry, dermatoscopy, dermoscopy, infrared, medical thermography

## Abstract

(1) Background: The authors developed a new non-invasive dermatological infrared macroimaging analysis technique (MacroIR) that evaluates microvascular, inflammatory, and metabolic changes that may be dermoscopy complimentary, by analyzing different skin and mucosal lesions in a combined way—naked eye, polarized light dermatoscopy (PLD), and MacroIR—and comparing results; (2) Methods: ten cases were evaluated using a smartphone coupled with a dermatoscope and a macro lens integrated far-infrared transducer into specific software to capture and organize high-resolution images in different electromagnetic spectra, and then analyzed by a dermatologist; (3) Results: It was possible to identify and compare structures found in two dermoscopic forms. Visual anatomical changes were correlated with MacroIR and aided skin surface dermatological analysis, presenting studied area microvascular, inflammatory, and metabolic data. All MacroIR images correlated with PLD, naked eye examination, and histopathological findings; (4) Conclusion: MacroIR and clinic dermatologist concordance rates were comparable for all dermatological conditions in this study. MacroIR imaging is a promising method that can improve dermatological diseases diagnosis. The observations are preliminary and require further evaluation in larger studies.

## 1. Introduction

Non-invasive techniques used in dermatological diagnosis is still an open research field. Although no available techniques can completely replace histopathological examination in lesion diagnosis, dermoscopy is simple and low-cost, and currently represents the complementary solution most used by specialists [1]. Polarized light dermatoscopy (PLD) allows better structure and lesion visualization below the stratum corneum [2,3] improving diagnostic accuracy from 60.9% to 68.1% in malignant melanomas [4], from 70.6% to 84.6% in squamous cell carcinomas in situ (Bowen) [5], and from 66.9% to 85% in basal cell carcinomas [6]. Dermoscopy is subclassified into: (a) entomodermoscopy, when referring to infections and infestations; (b) inflammatory, to inflammatory disorders; and (c) pigmentation, to pigmentation disorders [7]. Inflammatory process visualization and dimensioning are some of the limitations. Therefore, better means to evaluate skin inflammation analysis is needed for improvement, to bring new objective data, and to reduce the clinical evaluation and dermoscopy subjectivity.

For this, a new category is presented: infrared macro imaging (MacroIR), a type of high-sensitivity infrared dermoscopy. All objects emit electromagnetic radiation, and most of the radiation emitted by the human body is infrared. An electromagnetic scanner is used to detect and quantify these waves emitted by the human body, which change in the presence of metabolic, inflammatory, vascular, and vasomotor changes in the skin and in the presence of inflammation. A combined use of dermoscopy and infrared thermography increased detecting inflammation specificity [8]. The aim of this study was to present different dermatological lesion cases comparing naked eye examination and conventional polarized light dermoscopy with infrared macro images patterns.

## 2. Materials and Methods

### 2.1. Equipment and Software

**Figure 1:**
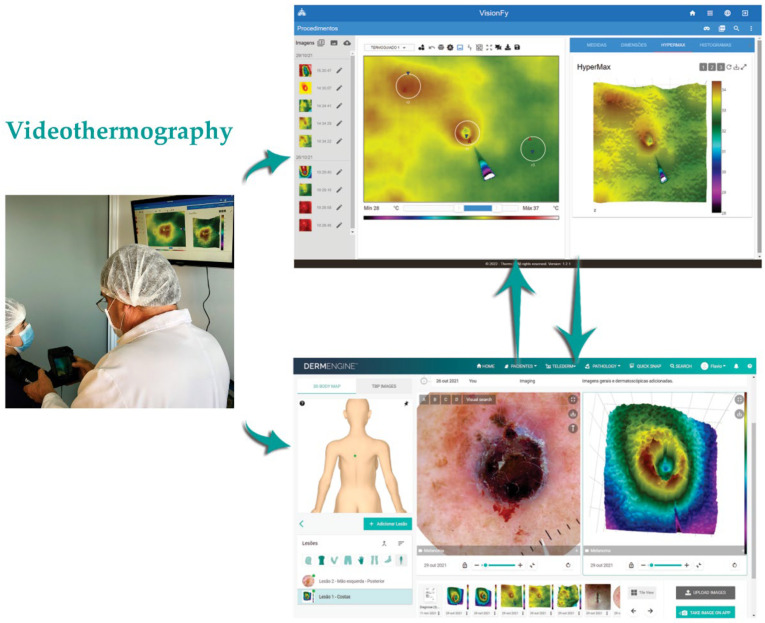
Videothermography using a TermoCam FLIR T530sc with Macro Mode lens adapter and specifics software VisionFy (Thermofy, Brazil) with Hypermax mode, and DermEngine platform (MetaOptica, Canada).

For image registration the following was used: (a) Dermlite DL4W-3GEN (3GEN, San Juan Capistrano, CA, USA) dermatoscope for dermoscopic and photographic images, with 24 white LEDs polarized and non-polarized infrared illumination, coupled to a smartphone (iPhone X-Apple 2018 256 GB); (b) TermoCam M530 infrared sensor (FLIR T530sc, Boston, MA, USA) for thermal imaging, 320 × 240 pixels resolution, 18.9 mm focal lens, 5/257 seconds exposure time, in macro mode, allowing to capture accurate temperature measurements in small targets without changing lens [9], calibrated using a Black Body Model BB-400, ISO 9001, CE IEC1010 certified, by Lutron Electronic; (c) A tubular rubber structure coupled to infrared camera lens, to obtain images at fixed distance without oscillations, at a perpendicular angle to analyzed area [10]. This technique is an adaptation of other techniques from the literature [11,12,13,14,15], that provides an easy way to capture same distance thermal images; and (d) Small sticky-paper arrows for more precise focus and target location. For image processing and analysis: (a) Dermoscopy images were obtained using Dermengine [16] platform (MetaOptica, Canada); (b) through videodermatoscopy, thermal images were transmitted to a computer in real time, exhibited by specific software for qualitative and quantitative analysis (VisionFy [17], Thermofy, São Paulo, Brazil), with alterations evaluated by measuring 3 Regions of Interest (ROI–R1, R2 and R3), the first two within lesion and the last at a distance; and (c) Hypermax mode (Thermofy, Brazil) use to spatial distribution study and better thermal patterns description. Cases were distributed into two groups according to its characteristics, for better understanding: malignant or borderline lesions; and benign lesions. Figure 1 demonstrates equipment and software usage.

### 2.2. Steps to Perform Image Registration and Analysis

**Figure 2:**
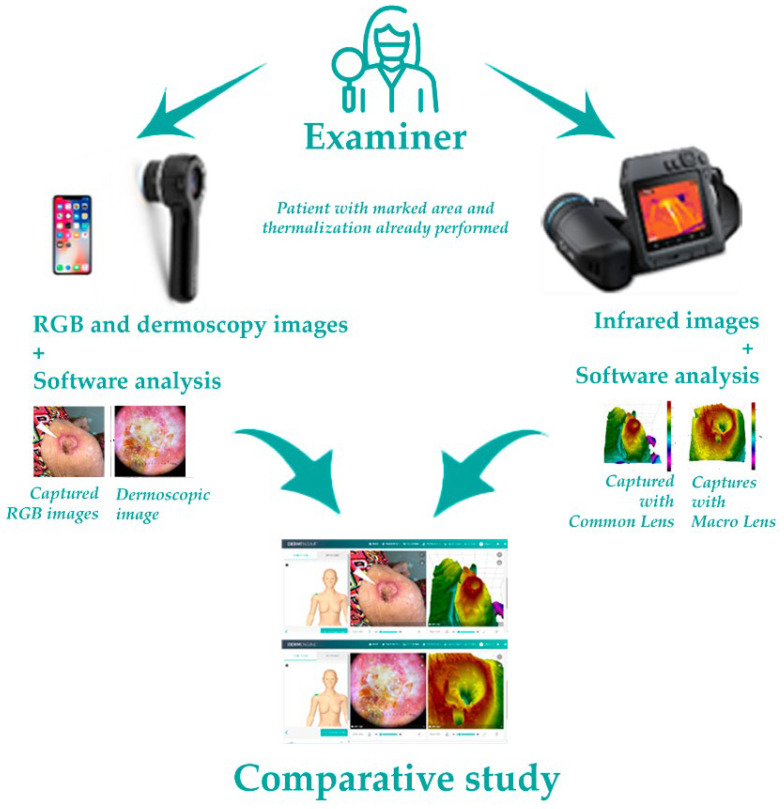
Schematic representation of the steps performed to capture and analyze imagens, both dermoscopic (DermEngine^®^) and thermographic (Thermofy^®^), and Hypermax^®^ creation.

The image registration proceeds as follows: (1) patient, after first clinical visual exam, waits 10 min in a room with temperature 21 °C ± 2 °C for thermalization [18,19], with the area to be studied exposed; (2) a marking is made with a small stick paper arrow that is placed near the lesion and pointing to it. This placement should preferably be performed using tweezers to avoid “thermal contamination”; (3) using the thermographic camera, with the lens in common mode, the first image is taken, at an average distance of 30 cm, which is stored on a memory card; (4) the camera is set to macro mode, and a tubular extension is attached to the sensor, to maintain a distance of 15 cm, and perpendicular position to the lesion, with the focus adjusted, using the tip of the arrow as a guide. The image is also stored on a memory card; (5) using a smartphone, RGB images of the lesion are collected and stored directly in the specific software (DermEngine^®^ v 5.62.1), after taking the thermographic images; (6) with the dermatoscope, coupled to the smartphone, dermoscopic images are also collected in RGB, with or without the use of gel to improve visualization, in simple mode or with polarized light, which are also automatically saved in the specific software (DermEngine^®^); (7) the thermographic images are transferred and treated by specific software (Thermofy^®^ v 1.2.1), in which the temperature measurements in ROIs are made, and the images created, using the Hypermax^®^ mode, in the same software; (8) these images are transferred to DermEngine^®^, where they are compared, complementing the diagnosis. These steps are illustrated in Figure 2.

### 2.3. Malignant or Borderline Lesions

**Figure 3:**
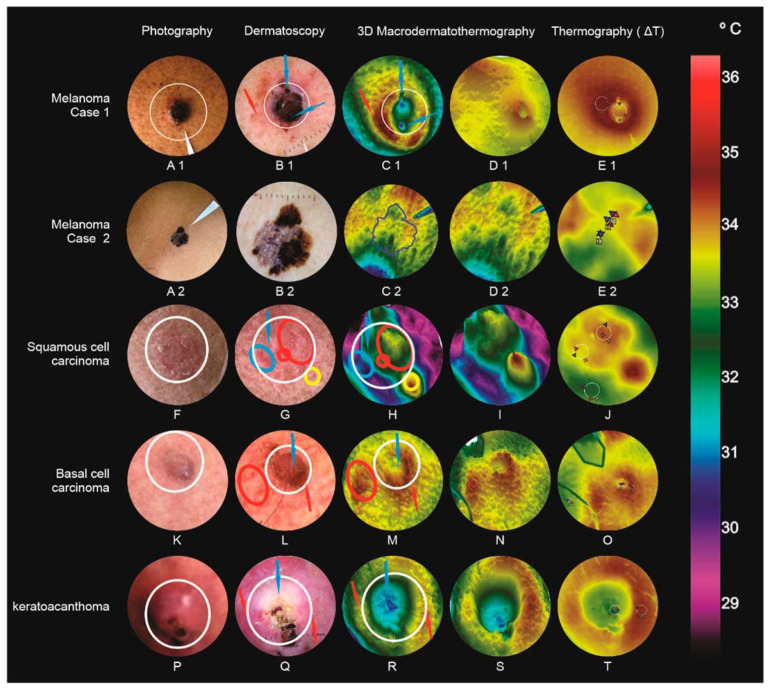
Malignant and borderline lesions. Melanoma case 1 (A1, B1, C1, D1, and E1), Melanoma case 2 (A2, B2, C2, D2, and E2), squamous cell carcinoma (F, G, H, I, and J), basal cell carcinoma (K, L, M, N, and O), and keratoacanthoma (P, Q, R, S, and T).

Five malignant tumors cases: 2 melanoma, squamous cell carcinoma, basal cell carcinoma, and keratoacanthoma (Figure 3).

### 2.4. Benign Lesions

**Figure 4:**
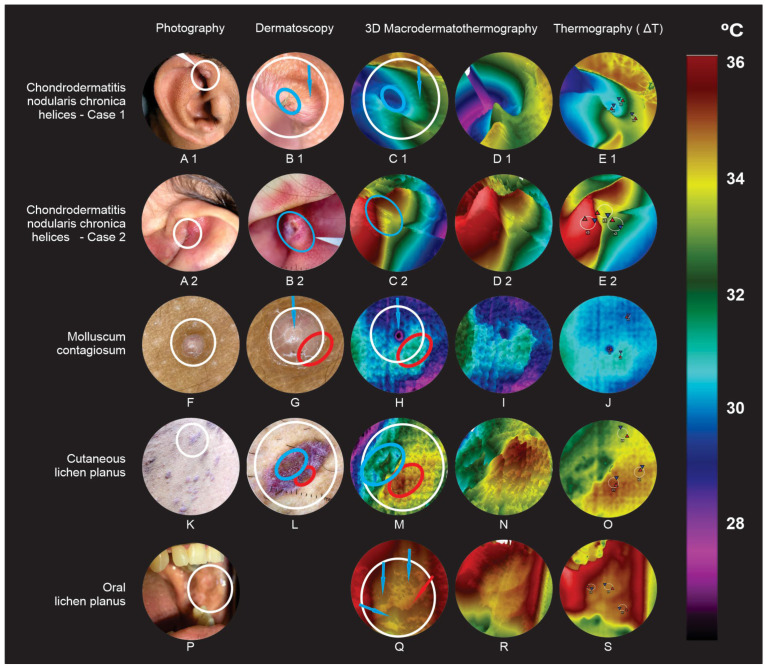
Benign lesions. Chronic nodular helix chondrodermatitis case 1 (A1, B1, C1, D1, and E1), Chronic nodular helix chondrodermatitis case 2 (A2, B2, C2, D2, and E2), molluscum contagiosum (F, G, H, I, and J), cutaneous lichen planus (K, L, M, N, and O) and oral lichen planus (P, Q, R, and S).

Five benign tumors cases: 2 chronic nodular helix chondrodermatitis, molluscum contagiosum, and lichen planus with two presentations, cutaneous and mucosa (Figure 4).

### 2.5. Evaluation Methods Comparison

Anatomopathological examination results were compared with clinical (visual), dermoscopic, and MacroIR evaluations. The anatomopathological result agreement with clinical observation was annotated (if there was agreement with anatomopathological result or not), in line with other studies [8,20].

## 3. Results

A standard lens infrared detector was used with an aperture angle of 24° and with macro mode activated, to reach a 71 μm point size without a need for lens change. At this point size, the transducer can accurately measure 0.03 °C temperatures, detecting small areas of 0.6 mm × 0.3 mm that show changes of temperature [9]. Through the software, it was possible to capture images with high magnification and small details in microenvironments, and quantify the data with greater precision and discrete thermal image variations. These infrared images were presented in a three-dimensional way to facilitate the physiological understanding and pattern description of the analyzed area.

### 3.1. Malignant or Borderline Lesions (Figure 3)

#### 3.1.1. Melanoma (Case 1)

A blackened nodule (8 mm × 5 mm) with superficial bleeding and a superimposed crust (left paravertebral interscapular region) (A1); Dermoscopy with brown amorphous structure, vascular pleomorphism, and reddish-milky areas (B1); MacroIR, irregular background (variable temperatures), crateriform formation (“*volcano sign*”), observed in bluish tone (blue arrows), surrounded by a raised and reddish halo (red arrow), extending to the upper left corner, like a tail (“*comet tail*”) (C1 and D1); ∆T_R2-R3,MAX_ = 1.62 °C (difference between the lesion’s most hyper-radiant area maximum temperatures, R2, and the external area, R3) (E1). Clark IV and 3 mm Breslow, in the vertical growth phase.

#### 3.1.2. Melanoma (Case 2)

An 8 mm flat lesion with varied colors, with black, gray, brown, and violaceous areas in the left supra clavicular region (A2); Dermatoscopy with a thick pigment network, abrupt endings in the periphery, irregular, heterogenic, and polychromic pigmentations, radial striations, pseudopods, and a grayish-blue veil; MacroIR: irregular background without major changes (C2 and D2); ΔT_R2-R3,MAX_ = 0.21 °C (difference between most hyper radiant area, R2, for the area outside the lesion, R3) (E2). Clark II/II and 0.5 mm Breslow, in a vertical growth phase with infiltration level.

#### 3.1.3. Squamous Cell Carcinoma

Left infraorbital, 6 mm × 7 mm, squamous erythematous plaque (F); Dermoscopy: actinic keratosis or Bowen’s disease, with background erythema, yellowish-white scale presence, and glomerular vessels. It is often impossible to clinically distinguish the two lesions [21,22], being considered by many authors as a same disease progression (G); MacroIR: irregularity, with two thermal elevations (yellow and green), with an enlarged base and crest (circled in red, glomerular vessels), and other thermal changes, as if they were plains and valleys (circled in blue). Outside the lesion perimeter, a new thermal elevation with a slightly narrower and more reddish crest than the previous ones, and a yellowish base, corresponding to a vessel without lesion interaction (circled in yellow, “*ridge sign*”) (H and I); ∆T_R1-R3,MAX_ = 1.58 °C (J).

#### 3.1.4. Basal Cell Carcinoma

Masseter, 3.6 mm in diameter, a red surface nodule, with pearly areas in the right region (K); Dermoscopy: basal cell carcinoma showed a segment with a central scar, visualized as pearly-white areas, and the presence of globules and arboriform vessels in the periphery [23], indicative of nodular subtype [24] (L); MacroIR: volcano cone-like structure with its central crater (blue arrow, central healing area), and greater peripheral metabolic activity (reddish volcano cone, red arrow), and some low “thermal mountains” on the periphery patterns, inside the red ellipse, (micro telangiectasia) (M and N); ∆T_R2-R3,MAX_ = 1.39 °C (O). Anatomopathological examination confirmed solid pigmented basal cell carcinoma.

#### 3.1.5. Keratoacanthoma

Right leg lateral region nodule, 2.2 cm in diameter, with raised, smooth, rounded edges, with a crateriform center, filled with hyperkeratotic plug (P); Dermoscopy: keratoacanthoma, showing central white rings, a centered homogeneous white area, seen as brownish-white, blood spots, and white regions without structure [25]. Polymorphic vessels were observed in the periphery, some glomerular vessels, others in staples, and some rectified vessels (Q); MacroIR: a large central depression in blue (blue arrows), surrounded by a varying heights mountains patterned chain (from green to red, red arrows), corresponding to those of dermoscopy of polarized light, and showing increased peripheral metabolic activity (R and S); ROI ∆T_R2-R3,MAX_ = 1.01 °C (T); Keratoacanthoma was confirmed by anatomopathological examination. These tumors have some clinical, epidemiological, and dermoscopic characteristics of their own, such as spontaneous regression [26], but they often resemble those of squamous cell carcinoma [25], and are therefore considered borderline.

### 3.2. Benign Lesions (Figure 4)

#### 3.2.1. Chronic Nodular Helix Chondrodermatitis (Case 1)

Right ear helix, 7 mm × 3 mm nodule, with rounded edges and discrete central keratotic area (clinical features such as keratoacanthoma) (A1); Dermoscopy: clear and irregular vessels on the outer edge (dermoscopic features such as keratoacanthoma) (B1); MacroIR: a deep crater pattern along the tumor’s entire length (lower temperature than the unaffected region), with edges superficializing as they approach like lesion limits, without achieving the same thermal elevation as healthy skin (blue ellipse and arrow) (C1 and D1); ∆T_R1-R3,MAX_ = −2.49 °C, an expressive and negative thermal ROI differences, capable to complement polarized light dermoscopy [27] (E1).

#### 3.2.2. Chronic Nodular Helix Chondrodermatitis (Case 2)-(Winkler’s Disease)

Left ear antihelix, 7 mm, exulcerated nodule with a central keratotic stopper (with keratoacanthoma clinical features) (A2); Dermoscopy: a rounded edge, with irregular linear vessels located, and a central ulceration (B2); MacroIR: deep crater that occupies almost the entire tumor extent, with its edges shallowing as it approaches the limits of the lesion (C2 and D2); ΔT_R2-R3,MAX_ = −3.82 °C (very significant and negative differences in thermal ROIs) (E2).

#### 3.2.3. Molluscum Contagiosum

Three mm whitish papules and central umbilication (F); Dermoscopy: vessels around the lesion (crown vascular pattern), with a central hole (G); MacroIR: distinct central crater, surrounded by thermal elevations at different levels, depending on small vessels that crown the lesion proximity (H and I); ∆T_R2-R3,MAX_ = 0.28 °C (J).

#### 3.2.4. Cutaneous Lichen Planus

Scattered brownish erythematous varying shapes and sizes papules and plaques (K); Dermoscopy: Wickham’s striae, with vessels in spots and areas with undefined brownish morphology [28] (L); MacroIR: blue plain with some thermal valleys pattern (corresponding to the brown amorphous area), a yellow plateau, and a central lesion region superimposed by a set of elevations, where a higher reddish color peak stands out, outward, corresponding to a greater blood vessels concentration (M and N); ∆T_R1-R3,MAX_ = 0.27 °C (O).

#### 3.2.5. Oral Lichen Planus

Plaques in an annular pattern on oral mucosa, a difficult dermoscopic evaluation region (P); although there are already defined dermoscopic patterns, oral lesion diagnosis is clinical and histopathological; MacroIR: Orange plateaus (blue arrows), surrounded by small reddish thermal elevations (red arrow) (Q and R); ∆T_R1-R3,MAX_ = 0.38 °C (S).

## 4. Discussion

Table 1 shows the infrared images’ formations summary, which are characteristic, and help in the faster diagnosis of skin diseases when compared with the already known dermoscopic structures. These are the thermal elevations that may represent increased metabolic activity and superficial vessels presence. A raised halo around a crater extending out from the tail-shaped lesion (“*comet tail sign*”) shows metabolic activity beyond the limits visible to the naked eye, and appear in the case of melanoma. Enlarged base and crest elevations may represent superficial vessels on infrared images that help distinguish an actinic keratosis from a squamous cell carcinoma in situ (Bowen’s tumor) with its glomerular vessels. Volcanic formations in infrared image can be explained by the central healing area and increased peripheral activity, which is very common in basal cell carcinomas. A large central depression, surrounded by a mountain range of different thermal elevations (“*valley sign*”) can be seen in keratoacanthoma. Deep thermal craters, which encompass the entire lesion, may be characteristic of chondrodermatitis, with vascular obstruction. Discreet hotter craters when centered and surrounded by small elevations may be a molluscum contagiosum characteristic. Thermal plains and plateaus patterns, surrounded by mountain ranges, were observed in lichen planus in both presentations’ lesions.

The reported temperature differences do not seem to correspond to other results. Some works mention temperature variations below 0.7 °C in malformations with high blood flow [29]. However, these works are about vascular malformations of large and small flow (Hemangiomas and lymph hemangiomas) that only reflect a more accentuated passage of blood in the region. Our study demonstrates neovascularization and the consequent intense metabolic increase, in line with previous work on metabolic increase, such as Gautherie’s [30].

Maximum temperature thermal difference analysis in varied ROI in each case showed that, in malignant or border lesions, there was a significant increase in temperature above 1.01 °C, in agreement with other thermographic studies [30] that show that the greater the thermal gradient, the worse the prognosis. On the other hand, there were minor positive differences below 0.38 °C in benign lesions, or significantly negative differences in the case of chronic nodular helix chondrodermatitis, perhaps due to the etiopathogenesis of this disease. All the temperature ranges measured in these cases are summarized in Table 2.

Future studies may bring continuous improvements to the technique, opening the possibility of determining thermal patterns that can increase diagnostic accuracy and decrease the use of excessive invasive techniques, such as biopsies, as well as adding new criteria to support imaging diagnosis.

Evaluating on a per case basis, and separating the lesions’ clinical aspects, the dermoscopic findings and MacroIR findings (including temperature differences), it is noted that they are not always compatible with the gold standard, which is histopathological examination. Diagnostic agreement between dermoscopy, MacroIR imaging, and clinical evaluation compared with histopathological results is summarized in Table 3. In 50% of the cases, they could receive a formal diagnostic only by clinical assessment, 40% could receive a diagnosis per dermoscopy evaluation, and 90% were consistent with histopathological diagnosis by MacroIR findings. Analyzing the elements found in each exam type, it is noted that the MacroIR complements the clinical and dermoscopic findings, providing greater security in the conduct being taken. Temperature differences between the evaluated areas play a fundamental role, as they show greater or lesser metabolic activity in the region with an increase or decrease in micro vascularization.

The structures formed in MacroIR image demonstrate, in a graphic and practical way, these differences, that can quickly aid clinical reasoning on per case basis.

## 5. Conclusions

MacroIR imaging is a non-invasive method, useful in the evaluation and monitoring of dermatological diseases by adding new patterns, and although it is not diagnostic, it can act as a valuable and easy-to-perform procedure to complement PLD and naked eye clinical examination. The concordance rates were comparable for all the dermatological conditions in this study. Due to the small number of cases, it has not yet been possible to determine definitive infrared patterns for different lesions. However, as an initial study, the provided information shown in this paper and the diverse literature on this topic demonstrate that MacroIR is a promising technique that can be used in dermatological assessments and has improved suspected diagnosis for the screening of benign, malignant, and borderline skin diseases, as it has been able to provide valuable information in 90% of the cases. Observations are preliminary and require further evaluation in larger studies.

## Figures and Tables

**Table 1:** Summary statement between the lesions studied with MacroIR and dermoscopic findings.

Case	Main Dermoscopic Findings	Dermoscopy Compatible, Incongruous, or Indifferent towards the Final Diagnosis	Main MacroIR Findings	MacroIR Compatible, Incongruous, or Indifferent towards the Final Diagnosis	Largest Temperature Differences (ΔT) Founded between Regions of Interest (ROIs)
Melanoma (Case 1)	- Brown amorphous structure - Vascular pleomorphism - Red milky areas	Dermoscopy left doubts with differential diagnosis with Basal Cell Carcinoma	- Irregular background (varied temperatures) - Crateriform formation in a yellowish tone, surrounded by a reddish halo that extends in the form of a tail.	ΔT and comet tail were points that helped to suspect more melanoma, aiding in dermoscopy	1.62 °C
Melanoma (Case 2)	- Thick pigment network with abrupt endings at the periphery - Irregular and heterogeneous pigmentation - Polychromies - Radiate striae, Pseudopods, and gray-blue veil	The structures found in dermoscopy were enough to indicate an excisional biopsy with high suspicion of melanoma.	- Irregular background without major changes (varied temperatures)	Lack of high ΔTs, as well as small surface changes, suggests “in situ” or superficial expanding melanoma. Anatomopathological examination results showed to be expansive superficial with thickness (Breslow = 0.5 mm)	0.21 °C
Squamous Cell Carcinoma	- Grouped glomerular vessels - Whitish yellow scales	In the first dermoscopy view, the vascular changes (glomerular vessels) that are very common in Bowen were not noticed. When evaluating the MacroIR, the changes led to a review of dermoscopy	- Irregular background - Two ridges in yellow and green of widened base and ridge - Plains and valleys	Visualization of the mountains in the MacroIR indicated the presence of vessels and forced a dermoscopy review.	1.58 °C
Basal Cell Carcinoma	- Arboriform vessels - Grayish blue dots and blood cells - Pearly white areas	Dermoscopy compatible with basal cell carcinoma	- “Cone of a volcano” - “Central crater”	MacroIR compatible with basal cell carcinoma	1.39 °C
Keratoacanthoma	- White circles in the center - Central homogeneous white area with white brownish areas - Blood stains - White areas without structure	Dermoscopy is compatible with keratoacanthoma, but it always leaves doubts in the differential diagnosis with Squamous Cell Carcinoma	- Great central depression - Range of mountains of varying altitudes surrounding the central depression	MacroIR compatible with dermoscopy and did not change the diagnostic hypothesis of keratoacanthoma nor the differential diagnosis with Squamous Cell Carcinoma	1.01 °C
Chronic nodular helix chondrodermatitis (Case 1)	- Fairly smooth rounded edge - Irregular linear vessels located on the edge - Central keratotic area	Dermoscopy very similar to keratoacanthoma	- Deep crater that takes up almost the entire extension of the tumor, with the superficializing of its edges as they approach the limits of the lesion.	MacroIR findings were consistent with Chronic Nodular Helix Chondrodermatitis, ruling out keratoacanthoma.	−2.49 °C
Chronic nodular helix chondrodermatitis (Case 2)	- Fairly smooth rounded edge - Irregular linear vessels located on the edge - Central keratotic area	Dermoscopy very similar to keratoacanthoma	- Deep crater that takes up almost the entire extension of the tumor, with the superficializing of its edges as they approach the limits of the lesion.	MacroIR findings were consistent with Chronic Nodular Helix Chondrodermatitis, ruling out keratoacanthoma. In this second case it was decisive in the diagnosis	−3.82 °C
Molluscus Contagious	- Central hole - Coronary vascular pattern	Compatible with the diagnosis of Molluscum Contagiosum	- Discreet central crater - Discrete elevations at different levels	MacroIR compatible with dermoscopy. Indifferent in the diagnosis of Molluscum Contagiosum	0.28 °C
Cutaneous lichen planus	- Wickham striae - Pots in points - Brownish areas without defined morphology	Dermoscopy compatible with the diagnosis of Lichen Plane Cutaneous	- Blank plain - Plateau in green - Set of elevations where a higher orange-colored peak stands out	MacroIR indifferent in the diagnosis of Lichen Plane Cutaneous Interesting to be able to follow the evolution after treatment	0.27 °C
Oral lichen planus	-	Dermoscopy was not performed due to technical difficulties	- Orange plateaus, surrounded by small reddish elevations.	MacroIR indifferent in diagnosis Compatible with the oral lesion and helps in the evaluation of the evolution after treatment.	0.38 °C

**Table 2:** Lesion temperature range, as measured in infrared images.

Diagnostic	Temperature Range (°C)	
	Minimum	Maximum
Melanoma (Case 1)	28.00	37.00
Melanoma (Case 2)	30.90	37.70
Squamous cell carcinoma	28.00	37.00
Basal cell carcinoma	28.00	35.40
Keratoacanthoma	28.00	34.80
Chronic nodular helix chondrodermatitis (Case 1)	28.60	37.00
Chronic nodular helix chondrodermatitis (Case 2)	26.20	37.00
Molluscus contagious	32.30	35.90
Cutaneous lichen planus	32.80	34.80
Oral lichen planus	28.00	37.00

**Table 3:** Diagnostic agreement between Dermoscopy, infrared macro imaging, and Clinical Evaluation compared with histopathological result.

Diagnostic	Was the Isolated Method Sufficient to Reach the Histopathological Diagnosis?		
	Clinical Evaluation	Dermoscopic	MacroIR
Melanoma (Case 1)			
Melanoma (Case 2)			
Squamous cell carcinoma			
Basal cell carcinoma			
Keratoacanthoma			
Chronic nodular helix chondrodermatitis (Case 1)			
Chronic nodular helix chondrodermatitis (Case 2)			
Molluscus contagious			
Cutaneous lichen planus			
Oral lichen planus			
**The isolated method was sufficient to reach the histopathological diagnosis (%)**	**50%**	**40%**	**90%**

Green when the test agrees with anatomopathological exam. Red when it does not.

## Data Availability

The data presented in this study are available on request from the corresponding author.
